# Effect of long-term administration of Cinnamomum cassia silver nanoparticles on organs (kidneys and liver) of Sprague-Dawley rats

**DOI:** 10.3906/biy-1805-103

**Published:** 2018-12-10

**Authors:** Kofi KOUAME, Aniekan Imo PETER, Edidiong Nnamso AKANG, Misturah ADANA, Roshila MOODLEY, Edwin Coleridge NAIDU, Onyemaechi Okpara AZU

**Affiliations:** 1 Department of Anatomy, College of Medicine, University of Lagos , Lagos , Nigeria; 2 Department of Anatomy, School of Medicine, University of Namibia , Windhoek , Namibia; 3 Discipline of Clinical Anatomy, School of Laboratory Medicine and Medical Sciences, University of KwaZulu-Natal , Durban , South Africa; 4 School of Chemistry and Physics, University of KwaZulu-Natal , Westville Campus, Durban , South Africa

**Keywords:** Histology, nanomedicine, toxicity, degenerative, congestion

## Abstract

This study investigated the toxic effects of silver on the kidneys and livers of Sprague-Dawley rats after administering multiple doses of silver nanoparticles synthesized using extracts of Cinnamomum cassia (CcAgNPs). Twenty-four Sprague-Dawley rats (250 ± 20 g) were randomly assigned to four groups (A-D) of six animals per group and treated for 8 weeks. Group A was administered 200 mg/kg of Cinnamon Cassia extract (Cc), group B 5 mg/kg of CcAgNPs, group C 10 mg/kg of CcAgNPs, and group D normal saline. Body weight was measured weekly and fasting blood glucose was measured fortnightly. At the end of the experiment, animals were euthanized and organs (livers and kidneys) were fixed in neutral buffered formalin and processed for light microscopy (H&E). Body weight differences were significantly higher (P < 0.05) in the low-dose Cc group and the kidney to body weight ratio was not significant. Renal function analysis of proteins and ketones showed a significant increase in CcAgNP-treated rats (P < 0.05). Kidney and liver histology showed distortions in hepatocytes and sinusoidal linings with infiltrations especially in the higher dose groups. Kidney histology mirrored degenerative changes in glomerular and Bowman's capsules with bfirillary mesangial interstitium. CcAgNPs impairs renal and hepatic morphology and function after a long period of administration.

## 1. Introduction

Silver nanoparticles (AgNPs) have gained unique attention because of their attractive properties, including their high surface to volume proportions, reactant properties, and antimicrobial impact (Okafor et al., 2013) . This is particularly relevant in the health sciences as it opens new frontiers in drug synthesis and delivery needed to target some of the sanctuary sites difficult for normal therapeutic doses of drugs to penetrate (Peter et al., 2018) . However,most techniques used for the synthesis of nanoparticles (NPs) are costly and may negatively influence biological systems. Therefore, green synthesis using plant materials offers a relatively more secure and ecofriendly methodology for NP synthesis and has been readily adopted for silver nanomaterials. Plants offer an attractive system for NP synthesis because of their capacity to deliver an extensive variety of optional metabolites with weak potential for toxicity. Plant extracts offer less biohazard, are environmentally friendly, and are less delicate to handle compared to microscopic organisms and therefore offer a green option for biosynthesis, such as AgNPs (Pandey et al., 2013) . It is pertinent to mention that some plants are already being exploited in this technique for green nanoparticle synthesis, including the leaves of Olea europaea and the bark of the Cinnamomum zeylanicum tree, used as a part of conventional prescriptions in Turkey and in other nations (Kumar et al., 2013) .

Cinnamomum cassia, also known as Chinese cassia or Chinese cinnamon, is an evergreen tree originating from southern China and widely cultivated there and elsewhere in southern and eastern Asia (India, Indonesia, Laos, Malaysia, Taiwan, Thailand, and Vietnam). In South Africa, mostly in the KwaZulu-Natal region, it is used as a spice and for medicinal remedy in various illnesses, such as diabetes. Lee (2002) earlier reported its antidiabetic property in vitro. Cinnamomum cassia extracts offer additional qualities for use in NP synthesis due to their phytoconstituents (phenolics and flavonoids), which act as capping agents providing stability to Ag nanoparticles with the ability to control the size and shape of NPs by giving extra layers to them (Ahmed et al., 2016) .

Plant-based AgNP utilization in medicine and pharmaceutical interventions may become the way to go owing to the availability, nontoxicity, multitargeting mechanism of action, and variety of metabolites that facilitate reduction of Ag ions in biological systems (Webster et al., 1992) . Cinnamon cassia AgNPs have been reported to be less toxic and also enhanced antiviral activity against the H7N3 influenza A virus (Fatima et al., 2016) . However, Yun et al. (2018) in a recent study revealed that high-dose intake of cinnamon extract (2000 mg/kg) showed potential nephrotoxicity and hepatotoxicity to both males and females as evidenced by obvious increases of kidney/liver weight along with a small but statistically significant elevation of total cholesterol level.

The study by Shakeel et al. (2018) , however, found that cinnamon extracts showed a significant ameliorative role in the antioxidant system in response to elevated levels of titanium dioxide nanoparticles or titanium dioxide bulk salt-induced oxidative stress with recovery of the antioxidant system as well as histological damages and some hematological parameters in rat livers treated with titanium dioxide nanoparticles or titanium dioxide bulk salt. There is therefore no consensus with respect to the safety of AgNPs or their conjugates (Connor et al., 2005; Khlebtsov and Dykman, 2011) , warranting further investigations.

Our laboratory has utilized various animal models to demonstrate potential interactions of plant-based adjuvants in many therapeutic conditions for diabetes (Ismail et al., 2017) , hypertension (Azu, 2015) , and alcohol-induced antiretroviral toxicity (Ogedengbe et al., 2018) , exploring various markers for biochemical, immunological, and histomorphometric studies. The aim of this study was to evaluate and report the histopathological and biochemical effects of CcAgNPs in male Sprague-Dawley rats after multiple oral administrations.

## 2. Materials and methods

### 2.1. Collection of plant material

Pure Cinnamomum cassia powder was purchased from Warren Chemistry Specialties (Pty.) Ltd., Cape Town, South Africa (reference 492733), during winter break, and silver nitrate (AgNO ) was obtained from Capital 3 Laboratory (Pty.), KwaZulu-Natal.

### 2.2. Preparation of Cinnamomum cassia (Cc) aqueous plant extracts

For the preparation of C. cassia aqueous extracts, approximately 10 g of the powdered plant material (bark) was added to 300 mL of double-distilled water and allowed to boil for 10 min at 45 °C according to the method of Shankar et al. (2017) . The resulting mixture was filtered and stored in a refrigerator at 4 °C until analyzed.

### 2.3. Synthesis of Cinnamomum cassia silver nanoparticles (CcAgNPs)

AgNO3 was added to C. cassia extract (100 mL) to form the AgNPs (Ali et al., 2016; Elobeid, 2016) . The formation of AgNPs was observed by a change of color (Shankar et al., 2015) .

### 2.4. Structural analysis of CcAgNPs

**Figure 1:**
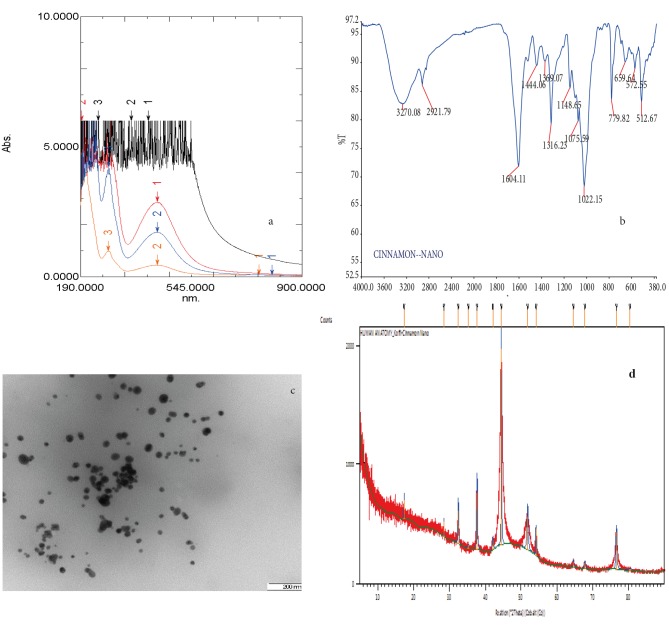
a) UV-Vis absorption spectra for Cinnamomum cassia silver nanoparticles (CcAgNPs), b) FTIR spectra of aqueous Cinnamomum cassia silver nanoparticles (CcAgNPs), c) TEM images of Cinnamomum cassia silver nanoparticles (CcAgNPs), d) XRD patterns of Cinnamomum cassia silver nanoparticles (CcAgNPs).

Figure 1a shows the ultraviolet-visible (UV-Vis) spectra of CcAgNPs, which are in line with previous results that reported that the UV-Vis absorption spectrum with a distinct peak at 445  nm indicated a surface plasmon resonance for AgNPs, ranging from 2 to 100  nm in size (Shahverdi et al., 2007) .

Fourier transform infrared spectroscopy (FTIR) showed an absorbance peak at 3270 cm–1 (OH stretch), 2921 cm–1 (C-H stretch), and 1604 cm–1 (C = C stretch), indicating the presence of polyphenols and conjugation in the extracts (Figure 1b). Our results displayed a peak at about 500 nm, which is supported by transmission electron microscopy (TEM) analysis revealing that the NPs are of smaller size (Figure 1c). The absorbance peak confirmed the formation of AgNPs.

Additionally, TEM (Figure 1c) images confirmed that the morphology of the CcAgNPs was highly variable with a variety of shapes (spherical, longitudinal, and irregular). Their size variations were from 12 to 42 nm. Nanoparticle behavior appears to be a function of size and shape. Moreover, X-ray difraction (XRD) (Figure 1d) analysis showed six distinct difraction peaks. The strong peak of silver (1646.63 and 44.693 nm) followed by two medium peaks at 468.56 and 310.62 nm indicated that the biosynthesized NPs were indeed made up of only silver.

### 2.5. Experimental animals

Twenty-four pathogen-free male Sprague-Dawley rats weighing 250 ± 20 g were selected for the experimental study. They were kept and maintained under laboratory conditions of temperature of 21.5–22 °C, humidity of 60 ± 1%, and a 12-h light/dark cycle. They were allowed free access to food (standard pellets) and water. Animals were allowed to acclimatize for 7 days in order to avoid causing stress during the experimental period. Experimental protocols and procedures used in this study were approved by the institutional ethics committee of the University of KwaZulu-Natal, ethics reference number AREC/074/016D.

### 2.6. Measurement of blood glucose

Thereafter, animals were kept under observation. Fasting blood glucose was measured on days 0, 14, 28, 42, and 56. Animals were tested using Roche Accu Chek Active 50 blood glucose strips (Dischem, South Africa), with blood collected from the tail vein of the rats at 1000 hours daily.

### 2.7. Experimental design

In this experiment, Sprague-Dawley rats were randomly assigned to different groups. The following groups of six animals each were treated. Group A comprised rats that received Cc (200 mg/kg), orally, once per day. Group B comprised rats administered a low dosage of CcAgNPs (5 mg/kg), orally, once per day. Group C comprised rats administered a higher dosage of CcAgNPs (10 mg/kg), orally, once per day (Sulaiman et al., 2015) . CcAgNPs were dissolved in normal saline and administered orally, once daily, and group D comprised rats (the control) that also received normal saline (1 mL). All administrations were done at 1000 hours daily for 56 consecutive days, through a rat gavage needle (Daisy and Saipriya, 2012). Body weight was recorded every week in the morning between 0800 and 1000 hours using an electronic balance (Zeiss, Germany; 0.000 g). After 56 days of administration, all animals were sacrificed using excess halothane anesthetic. Blood was then collected via transcardial puncture for biochemistry. Liver and kidney tissues were harvested after a laparotomy and processed for light microscopic studies. In addition, the weights of each kidney and liver were recorded.

### 2.8. Histopathological examination of kidney and liver tissues

Organs were washed in saline and fixed in 10% neutral buffered formalin for 24 h. Samples were transferred to 70% ethanol (Latendresse et al., 2002) . They were then processed using ascending grades of ethyl alcohol to dehydrate the samples, and xylene was used as the clearing agent. Samples were then mounted in molten Paraplast at 58–62 °C; slices of 4–5 μm were cut using a microtome (HM 315 microtome, Walldorf, Germany) from the prepared blocks and stained with hematoxylin and eosin (H&E). Sections were viewed and photographed using an Olympus light microscope (Olympus BX51, Olympus Optical Co. Ltd., Tokyo, Japan) with an attached camera (Olympus E-330).

### 2.9. Statistical analysis

All results are presented as the mean ± standard deviation of the mean. Statistical investigations were done utilizing one-way analysis of difference (ANOVA) followed by Tukey’s post hoc tests utilizing Graph Pad Prism Version 5. This allowed statistical comparison between the control and treated groups and statistical significance was acknowledged at P < 0.05.

## 3. Results

### 3.1. Effect of CcAgNPs on weight of body and vital organs

The oral treatment of rats with low and high doses of CcAgNPs (5 mg/kg and 10 mg/kg) impacted the body weight of experimental animals compared to the control as well as those treated with Cc. Body weight of experimental rats treated with CcAgNPs at low doses increased significantly compared to those treated with high doses and the control (P < 0.05) (Table). In addition, weights of kidneys and livers of rats were not different between those treated at low doses of CcAgNPs and the control as well as the group treated with Cc and this was not statistically significant at P < 0.05.

**Table:** Effect of Cinnamomum cassia silver nanoparticles (CcAgNPs) on weight of body and vital organs (mean ± SD, in mg).

Group	BWi	BWf	BWd	KW	BWKR	BWKRI	LW	BWLR	BWLRI
A	220± 6.6	220 ± 10	0	1.9 ± 0.14	0.89 ± 0.029	8.9	8.4 ± 0.70	0.0038 ± 0.002	0.38
B	310 ± 13	380 ± 17*	70. ±00	2.2 ± 0.67	0.005 ± 0.0003	0.5	12 ± 0.49	0.032 ± 0.003	3.2
C	310 ± 15	350 ± 23	40±.00	2.1 ± 0.08	0.006 ± 0.0006	0.6	12 ± 0.36	0.034 ± 0.002	3.4
D	320 8	360 ± 17*	40 ±00	2.4 ± 0.11	0.0067 ± 0.0003	0.67	13 ± 0.43	0.036 ± 0.021	3.6

*: Statistically significant (P < 0.05). Group A – rats treated with Cc, Group B – rats treated with low doses (5 mg/kg) of CcAgNPs, Group C – rats treated with high doses (10 mg/kg) of CcAgNPs, and Group D – control. BWi = Initial body weight, BWf = final body weight, BWd = body weight, KW = kidney weight, BWKR = body weight/kidney ratio, BWKRI = body weight/kidney ratio index, LW = liver weight, BWLR = body weight/liver ratio AND BWLRI = body weight liver ratio index.

### 3.2. Effect of CcAgNPs on blood glucose

**Figure 2:**
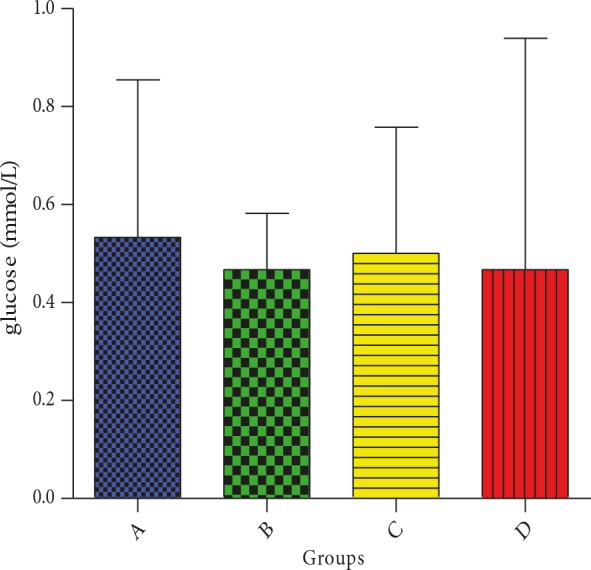
Effect of Cinnamomum cassia silver nanoparticles (CcAgNPs) on blood glucose of Sprague-Dawley rats. Group A – rats treated with Cc, Group B – rats treated with low doses (5 mg/kg) of CcAgNPs, Group C – rats treated with high doses (10 mg/kg) of CcAgNPs, and Group D – control. Not statistically significant at P < 0.05.

It was observed that fasting blood glucose levels of control animals were essentially similar to those of treated animals. Animals in groups B and C treated with CcAgNPs (5 mg/ kg and 10 mg/kg, respectively) showed the same levels of fasting blood glucose (Figure 2).

### 3.3. Effect of CcAgNPs on renal function parameters

**Figure 3:**
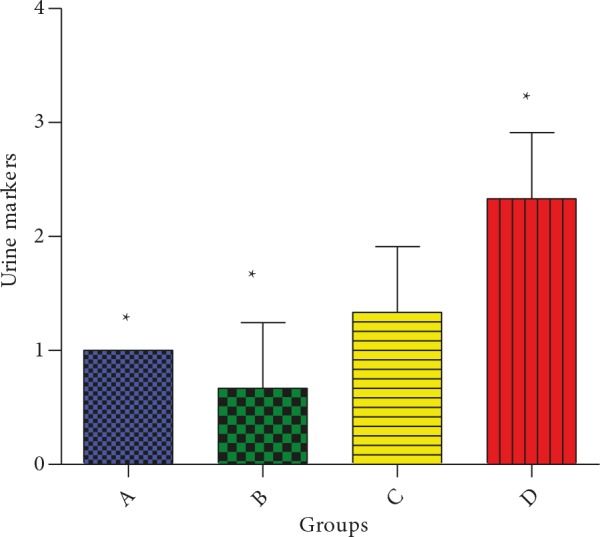
Effect of Cinnamomum cassia silver nanoparticles (CcAgNPs) on renal function parameters of urine proteins, ketones, and hemoglobin markers in Sprague-Dawley rats. *: Statistically significant (P < 0.05). Group A – rats treated with Cc, Group B – rats treated with low doses (5 mg/kg) of CcAgNPs, Group C – rats treated with high doses (10 mg/kg) of CcAgNPs, and Group D – control.

It was observed that protein, ketone, and hemoglobin levels in the urine of control animals were lower than those of treated animals. Animals in group B, treated with low doses of CcAgNPs (5 mg/kg), showed a moderate presence of protein, ketone, and hemoglobin in the urine, while those in group C, treated with high doses of CcAgNPs (10 mg/kg), displayed considerable presence of urine markers (Figure 3).

### 3.4. Effect of CcAgNPs on the morphology of livers in

**Figure 4:**
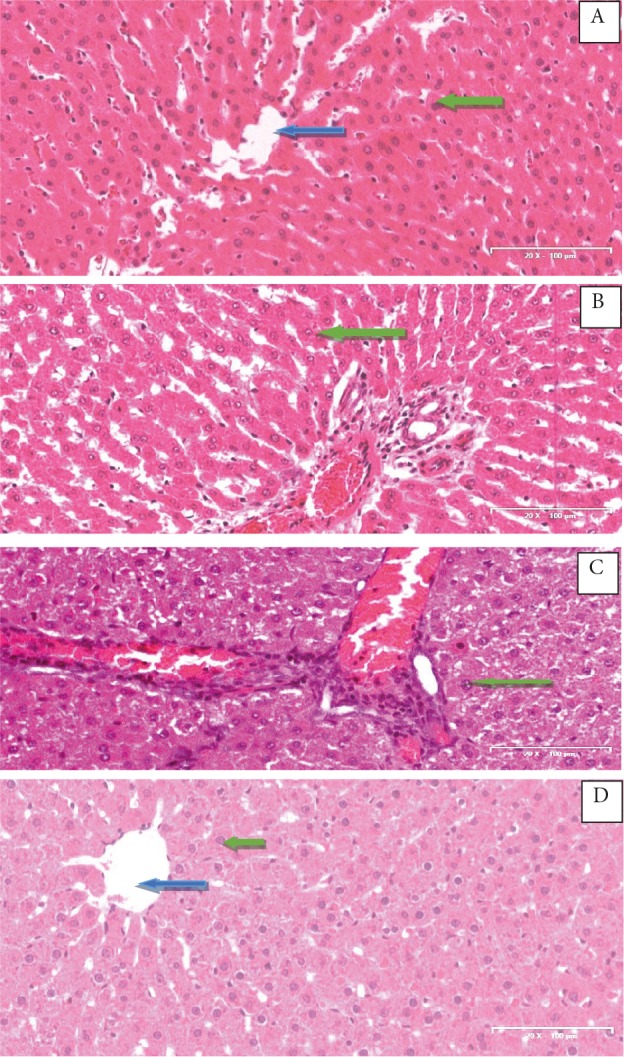
Effect of Cinnamomum cassia silver nanoparticles (CcAgNPs) on histological profile of liver in Sprague-Dawley rats. Group A – rats treated with Cc, Group B – rats treated with low doses (5 mg/kg) of CcAgNPs, Group C – rats treated with high doses (10 mg/kg) of CcAgNPs, and Group D – control. Blue arrow: central vein; green Arrow: hepatocytes.

Histological sections of liver tissues of rats were prepared and stained with the standard H&E technique and are presented in Figures 4A-4D. Figure 4A shows a liver section with outlines of hepatocytes showing nuclei that are clearly visible while the central vein appears distorted with loss of sinusoidal lining. Histologic sections of rats treated with 5 mg/kg of CcAgNPs (Figure 4B) show a distorted cytoarchitecture of hepatocytes, widened sinusoidal spaces, and focal congestion in the central veins. There are also infiltrations in the sinusoidal spaces. Rats treated with 10 mg/kg of CcAgNPs (Figure 4C) showed severe and generalized distortions in hepatocellular arrangement with nuclear condensation and pyknosis and areas of vacuolar changes suggestive of loss of liver architectural support/or bifrosis. Sections of the control rats (Figure 4D) essentially show normal hepatocytes lining sinusoidal spaces with arrays of radiation towards the central vein. Hepatocyte nuclei are clearly visible and well stained.

### 3.5. Effect of CcAgNPs on the morphology of kidneys of

**Figure 5:**
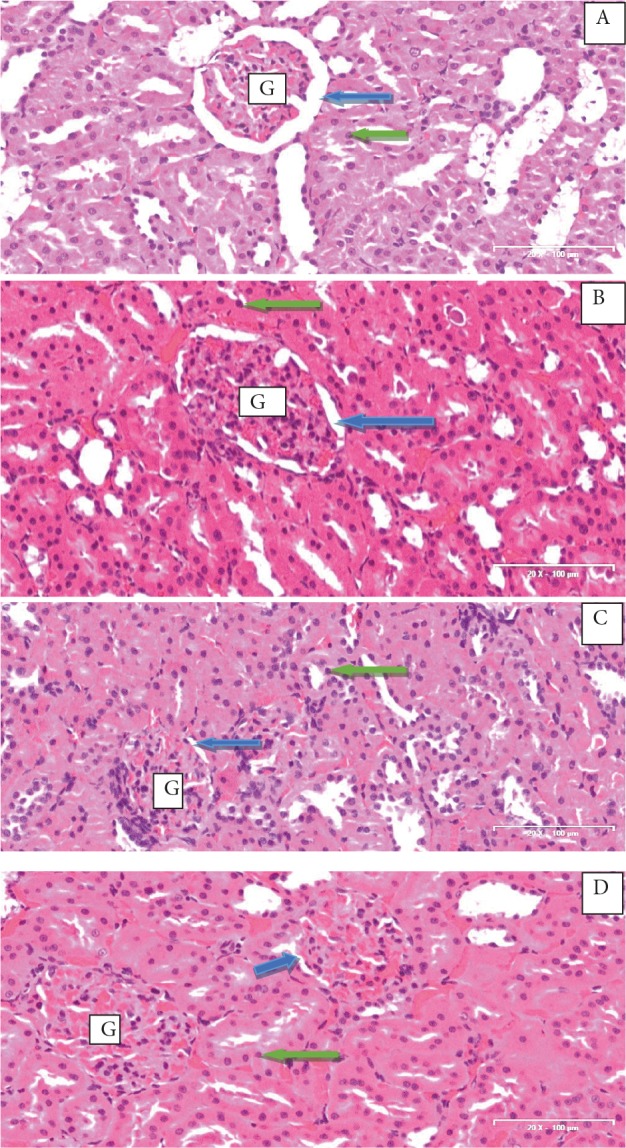
Effect of Cinnamomum cassia silver nanoparticles (CcAgNPs) on the histological profile of kidneys in Sprague- Dawley rats. Group A – rats treated with Cc, Group B – rats treated with low doses (5 mg/kg) of CcAgNPs, Group C – rats treated with high doses (10 mg/kg) of CcAgNPs, and Group D – control. G: Glomeruli; blue arrow: Bowman’s space; green arrow: nuclei.

Histological sections of kidney tissues of rats in the group treated with Cc (Figure 5A) showed the outline of renal corpuscles with glomeruli and Bowman’s capsular space distinctly showing mild atrophy of glomeruli. The nuclei of collecting tubules are identifiable and mesangial materials are delineated, showing areas of vacuolar changes. Sections from the kidney of rats treated with 5 mg/kg of CcAgNPs (Figure 5B) exhibited Bowman’s space and glomerular tissue congestion suggestive of necrotic changes. The mesangial tissues also showed nuclei of tubules that appeared deeply stained and interspersed. There was generalized atrophy of glomeruli with bfirillary meshwork in kidney tissues of rats treated with 10 mg/kg of CcAgNPs (Figure 5C) compared to the control (Figure 5D). In the control group (Figure 5D), histological sections appear essentially normal with glomeruli and Bowman’s capsular spaces depicting clear outlines with no detectable pathology. The mesangium is essentially preserved with nuclei of tubules visible and normal.

## 4. Discussion

While the utilization of AgNPs for biomedical purposes continues to grow, our insights and understanding of their impacts on living cells and on biochemical structures remains insufficient (Adeyemi et al., 2015; Sulaiman et al., 2015) . In this study, we explored the effects of varied doses of CcAgNPs on the livers and kidneys of SpragueDawley rats following oral treatment. Although animals treated with CcAgNPs showed no obvious signs of distress during the experimental period, they appeared to be less active and alert compared to the control group. There were no clear differences between the skin colors of the groups. Abdominal palpations did not reveal any abnormal mass, which was confirmed at autopsy.

Data from this study report changes in the body weight and organ/body weight ratios. These changes in organ/ body weight ratios may be suggestive of toxicity subsequent to the administration of CcAgNPs, as corroborated by previous researchers who revealed that NPs altered body and organ weights considerably (Sulaiman et al., 2015) . Our results contrast with the reports of Song et al. (2017), which showed that Cc extract administration significantly decreased body weights, food intakes, and serum levels of glucose in obese mice.

While Cinnamomum cassia is commonly been used for weight control in traditional medicines, this discordancy may likely be attributed to the diverse experimental design inherent in the two studies as well as other possible mechanistic pathways not clearly understood. Similarly, aqueous extracts from cinnamon have been shown to increase in vitro glucose uptake and glycogen synthesis alongside increased phosphorylation of the insulin receptor. These overall effects are likely to aid in triggering the insulin cascade system with potential hypoglycemic effects (Jarvill-Taylor et al., 2001) . Our results did not show any significant changes in blood glucose levels between the control group and treated animals, and neither did CcAgNPs.

As far as the histopathological studies were concerned, they showed degenerative changes and atrophy in renal and hepatic tissues of the rats treated with CcAgNPs in this study and this was exacerbated in the group receiving higher dose of CcAgNPs. Recent studies point to the potential of NPs aggregating in tissues such as the liver, which may provoke such observed alterations (Sulaiman et al., 2015) even in liver cells of rats (Hussain et al., 2005) .

While some authors proposed that AgNPs decrease the action of mitochondria, which causes the reduction of accessible vitality for cells (Hussain et al., 2005) , it is also possible that damage to the hepatocytes could be attributed to absorption of silver after oral administration (Furchner et al., 1968) . More so, Cc is reported to be used for the treatment of diabetes, attributable to the phenolics contained therein, and it is possible that its beneficial role in diabetic nephropathy treatment is plausible.

For renal markers of injury (ketones, proteins, and albumin) as well as qualitative histological evaluations, these derangements were more prominent with the higher dose of CcAgNPs. In agreement with our study, other studies reported toxic side effects on the renal tissue, which subsequently impacted renal function (Vasanth and Kurian, 2017) , but this does not agree with studies by Luo et al. (2013) and Yan et al. (2015), possibly due to the fact that refined active compounds were tested directly or perhaps due to the differences in the experimental design as some were tested on animals in diabetic states.

Although our study did not report antioxidant markers, Cc has been reported to be rich in type A polyphenols, demonstrated to be responsible for improvements in fasting glucose even in clinical trial subjects (Anderson et al., 2008) . The extract has also shown a significant ameliorative role in the antioxidant system in response to elevated levels of titanium dioxide nanoparticles or titanium dioxide bulk salt-induced oxidative stress with restoration of the histological damages in rat livers treated with titanium dioxide nanoparticles or titanium dioxide bulk salt (Shakeel et al., 2017).

 In conclusion, our findings revealed that biosynthesized silver nanoparticles using extracts from Cinnamomum cassia (CcAgNPs) caused injury to vital organs such as the liver and kidneys in normal healthy rats. The toxic effects appear related to the internal deposition of AgNPs, which in turn appeared dose-related. It is recommended that detailed immunological and electron microscopic evaluations of the tissues be carried out alongside other markers of liver/kidney injuries (biochemical assays for enzymes) in order to clearly establish specific alterations according to the dosage of CcAgNPs used.

## Acknowledgments

We acknowledge the College of Health Sciences, UKZN, for financial support to doctoral student Kofi Kouame. This work was supported in part by grants of the National Research Foundation of South Africa to the senior author (OOA; Grant U99053) and to Dr Roshila Moodley (Grant 94041). We also thank the School of Chemistry and Physics, UKZN (Westville Campus), and especially the support of Judie Magura and Bongisiwe Shelembe. The authors also acknowledge the UKZN Nanotechnology Platform.
